# Did malaria elimination begin to lose its way in 1925? “If you think education is expensive, try ignorance.”

**Published:** 2022-12-20

**Authors:** Anton Alexander

**Affiliations:** 1 BC Business Centrum, Elscot House, Arcadia Avenue, London N3 2JU, UK.

## Abstract

This paper begins with a brief examination of the first start made anywhere of a successful national malaria-elimination campaign. This start was made in 1922 in Palestine. The paper examines the essential education that was required to make the campaign so successful, thereby ensuring all inhabitants treated all aspects of the malaria-elimination as an absolute priority. Such priority led to the vital cooperation required for the necessary steps in the malaria-elimination method. But the paper also highlights a criticism of the campaign by the League of Nations in 1925 when the League sent its Malaria Commission to Palestine to investigate the campaign which it had heard about. The author tends to conclude that the education of all the inhabitants, of both Arabs and Jews, and which resulted in the inhabitants’ very strong cooperation, was actually contrary to, or in conflict with, the natural inclination of the members of the Malaria Commission whose governments were mainly still, in 1925, colonial powers.

The paper then moves on to present times and concludes the lack of success in malaria-elimination in many areas throughout the world is greatly due to the failure to provide that same personal education to the inhabitants that was provided in Palestine 100 years ago, principally because the governments in many malarious countries have not moved on from colonial times. The author’s personal conclusion, impression and opinion is that there appears to be hardly any sense of priority for the various malaria-elimination campaigns being presently conducted around the world, and where involved governments are probably still retaining old colonial attitudes when dealing with their respective populations.

## Background

**Figure 1.**
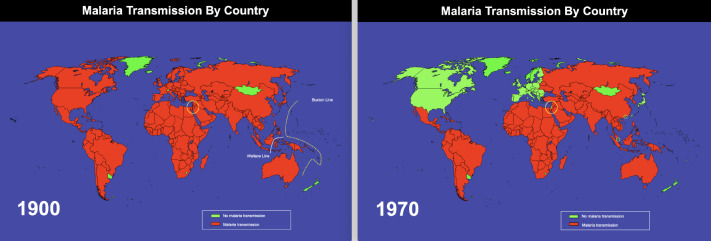
1900 and 1970 malaria maps showing malaria-free countries (in green) at those dates. Palestine was within the yellow circle on the 1900 map, and only Israel was shown as green within the yellow circle on the 1970 map.

This paper begins by reminding it is now 100 years since 1922 when the first start anywhere was made of a successful national malaria elimination campaign. That first start was made in Palestine and it was conducted without reliance on drugs, vaccines or mosquito bednets. The malaria elimination campaign began in 1922 and after 45 years, the area was declared free of the disease in 1967. The Global Health Group malaria maps [1] dramatically illustrate this outcome (Figure 1).

Malaria was severe in Palestine 100 years ago, the country was drenched in the disease and was accordingly uninhabitable in many areas. Over the centuries, Palestine had become desolate and neglected in many regions, sparsely inhabited, almost empty. The severity of the disease was such that it caused the collapse of half the British Army in Palestine in the final year of World War 1, in 1918, but fortunately for the British, the collapse occurred only days after its decisive defeat of the Turkish Army. Indeed, Palestine had been described as ‘*one of the most highly malarious countries in the world*’ [2] by Dr. Manson-Bahr, a then-officer with the British Army in 1918 in Palestine and who later was to become a Director of the London School of Hygiene and Tropical Medicine.

The 1922 Palestine Census [3] revealed the rural/ village inhabitants, numbering only 389,534 for the whole country, were the majority out of a sparse population totalling 757,182 (103,331 in Bedouin/ tribal areas and 264,317 in municipal/town areas). The total population in 2020 for the same area was approximately 13.5 million – an increase of almost 18 times the 1922 level.

### The original malaria-elimination method

In 1920, Dr. I. Kligler, a brilliant Public Health Scientist, a Jewish Zionist, had arrived to settle in Palestine, and was to become the architect of the subsequent malaria elimination campaign.

Kligler wrote of the difficulty of understanding at first sight how a country as sparsely settled as Palestine could have such a disease so widely spread and epidemics so continuous. He wrote that epidemics are usually correlated with crowding. He continued that the answer was furnished by a study of the socio-economic conditions prevailing there, and that because the country was small and undeveloped, there was a constant active movement of the various population groups including the Bedouin and those on annual pilgrimages. He noted that this movement was as effective in spreading malaria and maintaining its epidemicity as it would be in the case of any other infectious disease [4].

Kligler’s plan for malaria elimination was to be principally focused on the well-trodden path of destruction of the breeding sites of the mosquito which carried the disease. But his proposed method also included engaging with the whole rural Palestine population (albeit this was very small) to eventually secure the cooperation of both Arab and Jewish local communities who would assist with and also maintain the anti-malaria works that Kligler intended would be carried out, and thereby ensure the mosquito did not return to that district. This new and fresh approach of Kligler was to seek malaria elimination through involvement of the population by culturally-sensitive education. Each and every inhabitant had to be taught why certain anti-malaria works and actions were essential, and until that inhabitant understood, **the lesson had to be repeated**.

Basically, malaria 100 years ago was treated as a local problem, and the basis of the method employed in Palestine was firstly destruction of all mosquito breeding sites to prevent or control malaria in a locality. Secondly, once the breeding site(s) in that locality had been thoroughly destroyed, the inhabitants —few as there were— through continuous, thorough and systematic larval source management, were relied upon to ensure those breeding site(s) remained unproductive for mosquitoes for years on end [5].

Kligler’s method must have been viewed as something very different and special and which was evident from reports of malaria experts in the field. In 1925, the League of Nations Malaria Commission had been informed of anti-malarial works then taking place in Palestine and the Commission came to inspect. The Commission’s subsequent Report [6] opened with a relevant first-impression, immediately emphasising the existence of only few inhabitants:

*“Palestine is a small country and, as a whole, thinly populated (…). The population at the end of 1924 was estimated at 803,000 persons, of whom 103,000 belong to nomadic Bedouin tribes.”*

The Report then continued by dealing with topics under the following headings:

1. Why our visit to Palestine was of such special importance.
2. Why has the anti-malarial work in Palestine been so successful?
3. What the anti-malarial work in Palestine can teach us.

and concluded with:

*“(…) the work done in Palestine, by destroying pessimism, raising hopes, (…) becomes a welcome and invaluable addition to practical malariology, and the men who carried it out can be regarded as benefactors not only to the Palestine population but to the world as a whole.”*

The President of the Malaria Commission, Professor Nocht, was so impressed, he separately commented at the end of the Commission’s stay/inspection in Palestine:

“*Palestine showed the fruits of an energetic and victorious campaign which would stimulate others to follow the methods there employed.”* And *“It was not the custom of the Commission to make comparisons but he would on this occasion, say that the interest that Palestine had provided was unsurpassed by that of any of their other visits [to other countries]. (…) the Commission would (…) greatly profit by its visit to Palestine, and the world would surely benefit by what they had seen there, through the medium of the League of Nations.”* [7].

But it was not just the League of Nations that had come to inspect. Also in 1925, Dr. F. Russell, director of the Rockefeller International Health Division had inspected the anti-malaria work in Palestine, and wrote: *"I do not know when I have seen better and more successful anti-malaria work than that which is being done in Palestine (…). The co-operation of the people with the authorities leaves nothing to be desired (…). It is an ideal way to carry out malaria work because it makes the population served by the anti-malaria measures participants in the project from the beginning; they then have a better understanding of the problem and will be more ready than they otherwise would be to take care of the necessary maintenance."*[8].

Kligler from the outset had to ensure certain work was carried out properly, and the 1925 League of Nations Malaria Commission Report [6] noted (page 35) that in particular, the destruction of the breeding sites was conducted **thoroughly and systematically and after an exhaustive preliminary survey**.

I discovered only scant material about who actually was responsible for and conducted the initial works that destroyed the breeding sites and which thereby controlled the malaria. Because malaria was always treated by Kligler as a local problem, with the local breeding sites accordingly being destroyed, the local inhabitants would thereafter have been expected to maintain the works and ensure these former breeding sites remained destroyed. Rather than rely on untrained personnel and to ensure the initial destruction of the sites was carried out thoroughly and correctly, the literature appears to suggest it was only carried out by experienced and qualified persons, probably under the direction of a central authority and including an entomologist. Once destruction of the sites had successfully been carried out, only then could the area be turned over to the existing or future inhabitants (who would have already been educated/trained by Kligler) and who would then assume responsibility for maintaining the anti-malaria works [9,10].

There seems to be no recognisable equivalence to Kligler’s method with today’s methods of attempts at malaria elimination. Today’s methods are usually not locally based and instead have tended to be more in the nature of attempts at malaria control through use of insecticide-treated nets (ITNs) and indoor residual-spraying (IRS), but these methods in recent years have met with disappointing progress which has slowed and stalled [11]. Today’s methods usually rely upon each of the individual inhabitants using a net correctly and accepting their houses to be sprayed, and any inspector is completely dependent on an account provided by each inhabitant as to how a net was used the previous night. There seems to be no check or concept of thoroughness in today’s methods, it is difficult to verify accuracy, so one wonders how progress towards malaria elimination can expect to be gauged if it is unknown whether mosquitoes or parasites have been eliminated in a locality.

Once the Palestine breeding sites had been destroyed, the local inhabitants would have been expected to assume responsibility for maintenance of the anti-malaria work to ensure these sites remained destroyed. The task that was set for each inhabitant had to be reasonably manageable as the emphasis of the maintenance was on it being carried out correctly and thoroughly, and for which task the inhabitant had been educated. Unlike today’s methods, the Palestine maintenance work could be visually checked to ensure it had been carried out properly.

The fact that the country was so sparsely inhabited, almost empty, probably emboldened Kligler to attempt what seemed to be such an ambitious project, so few inhabitants making the task of educating each inhabitant that much easier.

Kligler wrote in his only textbook on malaria: “*The education of the inhabitants was (…) by no means the least important element which conditioned the success of the work. Without active co-operation on the part of the people, the work would have been only partially successful. It was possible to obtain their active co-operation only after they understood fully the significance and value of the work.”* [4].

In 1930, Kligler commented that malaria elimination involved education, being a more difficult task than only basic control of the disease, and explained further why education was so important. “*It was necessary to remove a variety of prejudices both on the part of the heterogenous population of the country and on the part of members of the medical profession. Moreover, the success of the work demanded the cooperation of various agencies —medical and colonising— whose passive resistance would have rendered the early work difficult and without whose active assistance full success would have been impossible.”* [12].

It is therefore surprising that Kligler did not write more extensively on how he set about obtaining the cooperation of inhabitants and dealing with the education to which he constantly refers. I, however, present below extracts from two papers, one by Kligler and the other by one of his colleagues, Dr. Abraham Levy, that provide a glimpse of the approach that was taken to secure that cooperation. This, presumably, was the approach actually witnessed by the League of Nations Malaria Commission when they inspected in 1925.

Kligler wrote in 1923:

*“(…) the first step was to examine **the entire population** in order to detect the [malaria parasite] carriers, and the second, to systemize and control the treatment (…) .”* [13].

*“The greatest emphasis was laid on measures against mosquito larvae (…).”* [13].

*“An important element (…) was the educational propaganda carried on along with the work [and which] started with a popular illustrated lecture on malaria, its causes, prevalence and modes of prevention.” “**During the examination the doctor spoke to each individual** explaining the purpose and importance of the [anti-mosquito] work. Throughout the year the malaria inspector would visit various delinquent families and impress them with the dangers resulting from their carelessness.” “For the first time, malaria assumed the importance of a real and preventable disease which should be eradicated.”* [13].

In 1933, the Egyptian Medical Association decided to hold its 6th Annual Congress in Jerusalem. A number of Kligler’s team were requested to provide papers on their malaria work for the event, and the following by Dr. Abraham Levy gave an insight into practical aspects relating to education:

*“(…) institute a systematic series of educational demonstrations to be presented by the sanitary inspectors when he makes his rounds. Instead of coming merely to inspect, to find fault or to look for trouble, **he should spend several days or even a week** among the inhabitants, to teach them by actual demonstrations the havocs that careless habits of needless spilling of water might bring to the district.” “It depends upon the ingenuity of the [sanitary] inspector to take his audience into his confidence and make them feel that he is only discussing the problem with them, rather than forcing his ideas upon them. This attitude will be more effective to carry out his rules and regulations than dozens of laws and threats. (…) The task is not easy. It may mean months of hard labour and many discouraging moments, but through perseverance and constant attempts, the results will come, and when they do, they will be of a permanent character.”* [14].

The few inhabitants in Palestine at that time were made up mainly of Arabs and Jews, many of the rural Jews being Zionists, as was Kligler himself. He realised he had to involve ALL the inhabitants, both Jews and Arabs, for malaria elimination to be successful, and for all the inhabitants to eventually see malaria elimination as a necessary common cause. Anti-malaria works and maintenance had to be conducted each year, and had to be conducted continuously, systematically and with great thoroughness. Nothing less would do. Nothing like this had ever been attempted before.

There was nothing new, nothing novel in the original destruction of the breeding sites. But the attention to the education was completely new.

In 1941, the British Mandate’s Department of Health had reviewed the malaria position in Palestine, and praised the co-operation:

*“In the true rural areas the lasting effects of swamp and marsh reclamation, the proper irrigation of gardens and similar areas, and the periodical canalisation, filling and clearing of stream beds, have so impressed the people by the resulting improvement in health, and the land thus made available to farmer and shepherd, that their prompt and energetic co-operation is one of the most remarkable features of the antimalarial campaign in this country.”* [15].

and also added:

*“As the general scheme has gradually advanced in scope, so the community self-help which has been stressed already as a particular feature of the antimalarial scheme here, has come more and more to the fore.”*

*“This is now a seasonal procedure after the April rains. (…) such cooperation was willingly, and even enthusiastically, given.”* [15].

As an aside and an interesting by-product of Kligler’s education was the comment in the 1925 League of Nations Malaria Commission Report:

*“Above all, it has succeeded in inducing the people of the country to take an interest in health problems and to cooperate in measures for the prevention of disease.”* [6].

In 1938, a British Government Commission reported *“(…) an abnormally high (and possibly unprecedented) rate of natural increase in the existing indigenous population.”* [16]. This fact had not in previous years generated great interest, but now the Commission Report continued by referring to the *“(…) astonishing change in the Arab population since [WW1] (…)”* and further added *“It would seem that the growth of population must be due mainly to a lower death-rate, brought about* (…) *by general administrative measures, such as anti-malarial control (…).”* The link of the extraordinary population increase to malaria elimination had been ever present. A previous 1937 Palestine Royal Commission Report had included: *“The growth in [Arab] numbers has been largely due to the health services, combating malaria, reducing the infant death rate, (…).”* [17]. This rate of increase in the population was not just a casual or passing local impression and was to be noted elsewhere. Thus Palestine, a previously almost-empty desolate country, was to experience an ‘abnormally high rate of natural increase’ and ‘astonishing change in the population’ which was confirmed in the Statistical Year Book of the League of Nations 1931/32 as **the highest in the world** [18].

### Criticism of the kligler method

So why didn’t the whole world embrace and adopt Kligler’s method, in particular his emphasis on education?

Because Palestine in 1922 was so thinly populated, literally empty in many places, Kligler’s team had the luxury of time to spend with each inhabitant. This approach was presumably something never before seen elsewhere by the visiting Malaria Commission. But the approach worked. It was in fact a form of community engagement at its very best, and the resultant cooperation of all the inhabitants, both of Jews and Arabs, eventually was to become a natural part of their daily lives for years and years, ensuring their involvement and contribution in the anti-malaria work.

Kligler’s attention given to all the inhabitants would, however, have been noted by the Malaria Commission members who must have thought it unusual and unorthodox. In view of the praise and compliments already mentioned, it was therefore surprising that the Commission would inexplicably include the following criticism in its 1925 Report:

*“It would be absurd to disparage the admirable work of [Kligler’s] unit, because its staff was provided with the means of carrying out the proposed schemes. Still I cannot help feeling that these considerable funds placed at their disposal tend to give these efforts more the character of a huge scientific experiment (such as might have been carried out in the year 1900, to test Ross’s theory) than of a practical anti-malarial campaign founded on an economic basis. It is certain that the Palestinian Government cannot follow on a large scale the lead given by [Kligler’s] unit on a small one, and I very much doubt whether any Government could ever do so.”* [6].

What on earth could have caused such a damning criticism following such praise, applause and compliments? What had disturbed the League of Nations to the extent that it had unexpectedly called forth such an outburst? The comment seems to imply there was an element of extravagance about ‘these considerable funds’ but there is also an impression that this is only a pretext for something else. This demanded investigation.

The Malaria Commission would already have been aware of the thousands of troops of the British Army in Palestine in the final year of World War 1 (in 1918). Under the direction of an entomologist, these troops had for six months during that year successfully controlled malaria in the area occupied by them by destroying mosquito breeding sites. The expense of the same task for Kligler in 1925 (but on a smaller scale) would, of course, have been expected by the Commission. Because the destruction of the breeding sites had ceased to be maintained by the British troops, the mosquitoes and disease had returned in 1918 only weeks after the defeat of the Turkish Army and the withdrawal of the British Army.

So, the only expense left that could have been unanticipated by the Commission and which would have gone towards making up ‘these considerable funds’ was the expense for Kligler’s education. If that is the case, it seems therefore that it was the existence of this expense alone which the Commission in 1925 used to rubbish Kligler’s method, in effect linking Kligler’s education to some new flaky theoretical exercise.

If this really was an attack on Kligler only for his education, by using expense as the weapon, the following response should be: if you consider education expensive, try ignorance. Using only expense as a weapon is shameful if attacking only the principle of education itself, particularly if the attack ignores what the education is all about.

It must be remembered that at that time, in 1925, the idea or concept of malaria elimination had never been feasibly considered anywhere else. Therefore, Kligler’s work would have appeared as a novel ‘huge scientific experiment’, and reference to presumably additional ‘considerable funds’ (for education) could easily have been presented as an extravagance.

If this is correct, then the Malaria Commission did immense harm by presenting the above comment in this way as the Commission appeared to be dismissing Kligler’s method —with its emphasis on real effective education for ALL the inhabitants— as of no consequence in malaria elimination.

What could have caused this outburst from the League of Nations? Perhaps any one of the following suggestions can provide an answer:

1. 1925 was still a colonial period, and Kligler’s individual attention to each inhabitant was probably not understood, possibly even uncomfortable for some of the Commission members, appearing extremely unusual and out of the ordinary to the Malaria Commission experts. Kligler’s education and corresponding intimacy with the inhabitants therefore for an anti-malarial campaign could have been unrecognisable to the Commission and viewed as unwanted, uncomfortable and an unwelcome precedent for those times … ; or
2. Because the population of Palestine was so small, a country that was almost empty, could the Malaria Commission have been genuinely suggesting that therefore Kligler’s efforts in Palestine just shouldn’t be taken seriously as a practical scientific demonstration? Could the Commission have been suggesting that Kligler was really treating Palestine as a laboratory, that the conditions did not reflect a common reality and that therefore the results should only be treated as purely theoretical? … ; or
3. Palestine in 1925 was only a sparsely populated backwater, and other than its religious connections, without any significance to the rest of the world. The Malaria Commission comprised the world’s cream of knowledgeable malaria scientists, and it may have been difficult for the ego to admit for some of them that a real breakthrough in malaria-elimination was taking place without an input from these experts.

For whatever reason, it would still have been very upsetting if the League of Nations was really suggesting there was no relevance or advantage whatsoever in educating the population for the purposes of malaria management. If that was the intention of the criticism, the League of Nations appears to have effectively warned-off countries from applying Kligler’s approach and would have thereby hampered progress towards malaria elimination in many countries by behaving so unhelpfully and pessimistically.

The Malaria Commission’s surprising 1925 rebuke may also have given an impression that Kligler was not only extravagant but also careless or cavalier with these ‘considerable funds’, and which impression would have been completely incorrect about Kligler. The 1923 Palestine Government Department of Health in its annual report [19] commented that Malaria Control Demonstrations had been conducted in various parts of Palestine to determine with what degree of efficiency and at what cost malaria could be kept in check or eliminated, in order that the possibility of large-scale malaria control in Palestine at a low cost might be established. Kligler was always conscious to seek ways of conducting anti-malaria work at as low a cost as possible, Kligler never considered the feasibility of an important project without first initially investigating the technical aspects and then costing those different aspects or possibilities.

The World Health Organization (WHO) Handbook on Integrated Vector Management [20] includes a brief historical note about malaria elimination. It merely states:

*"Before the Second World War (WW2), vector control was conducted predominantly by environmental control of the proliferation of mosquitoes.”*

*“There is evidence that environmental management had a clear impact on disease; however, [other than in Palestine], elimination of disease was never on the agenda. The advent of DDT and other organochlorine pesticides during the 1940s changed this situation.”*

*“The focus of vector control on insecticides meant that environmental management and other alternative methods were underexploited **or even forgotten**."*

The WHO is correct in suggesting the pre-WW2 methods were forgotten. The idea of real effective education of the inhabitants could have been a considerable asset in malaria elimination but would easily have been trashed by the 1925 comment of the League of Nations. Whatever the reason for the League’s comment, an appreciation of the importance for real effective education in respect to malaria elimination still has yet to recover from the damage caused to it.

Did the League of Nations have sympathies with colonialism? Did Kligler’s education represent a threat to the colonial powers? The puzzle is why the League of Nations saw fit to attack Kligler in this way.

But the fact remains that, other than in Palestine, Kligler’s successful malaria elimination method, in particular his emphasis on effective education, appears to have remained without national implementation anywhere.

### Modern times

The significance of cooperation in malaria elimination was effectively and succinctly explained as a necessity in two Ifakara Health Institute Master Classes in 2021 given by Professors Christian Lengeler [21] and Marcel Tanner [22], both acclaimed and highly experienced scientists dealing with malaria. Professor Lengeler of the Swiss Tropical and Public Health Institute in Basel, Switzerland concluded a Master Class in June 2021 on the topic of mosquito bednets with the comment that 20% of solutions to malaria elimination involved technology, knowledge and science, and that the remaining 80% of solutions involved ‘Improved management and better engagement with the Community’. Professor Tanner was Director of the Swiss Tropical and Public Health Institute from 1997 to 2015 and is now President of the Swiss Academy of Sciences. During a Masterclass in August 2021, he was asked for his view on the 20:80 solution, and he commented that 80% was the correct weight in his opinion. He added that the magic bullet on its own was harmless, but that the bullet became efficacious only when used with the 80% magic gun of a health and social system to bring it to, and for it to be accepted by, the people. The scientific opinion has therefore recognised that 80%, indeed the major part, of the malaria elimination solution is bound up with engagement with the community.

By introducing percentages/proportions, the Master Classes helped visualise the ‘weight’ of non-science/non-technology aspects when considering malaria elimination. It doesn’t matter that some may disagree with the percentages/proportions, the principle may still nevertheless be grasped that the popular technology, knowledge and science, whilst very important, is not the only part of the problem.

Whilst cooperation by the inhabitants in malaria elimination is today theoretically recognised as a necessity by the malaria community, the problem discussed today within the malaria community is how to practically achieve that cooperation.

An examination of present-day ‘struggling’ attempts at malaria control or elimination demonstrates the wide gulf that now exists between those countries endeavouring to obtain their respective populations’ cooperation and the successful cooperation achieved by Kligler all those years ago. Today’s methods appear to merely provide encouragement e.g. to use a bednet correctly (or even to use them at all). Today’s methods tend to merely emphasise ‘awareness’ of the disease. Theoretically, greater resources for education would, no doubt, encourage a greater use of bednets, as malaria elimination is more likely to be achieved if every inhabitant makes proper use of the bednets. But present methods of malaria elimination tend not to easily lend themselves naturally to the cooperation that Kligler would have planned. Perhaps repeated individual attention to each inhabitant would likely improve the take-up and correct use of bednets. Because of the nature of the elimination methods today (nets and spraying), the choice is left to the inhabitant whether or not to cooperate. In Kligler’s day, the education was pitched in such a manner that compliance with the tasks could be more readily checked, but that is not possible with today’s methods. Importantly and significantly, Kligler’s education was individually explained at an inhabitant’s level and repeated if necessary or requested, thus ensuring the inhabitant understood the necessity of the maintenance, and that thoroughness in the maintenance work was the only way to defeat the disease.

I thought it would be helpful for the reader to see an illustration of current approaches to malaria elimination, and below is an example of one such approach. I would wish in particular to draw the attention of the reader to the subsequent evaluations at the end of the example, the evaluations suggesting there is something missing from such an initiative. The attempt appears to be trying to create a habit of a correct nightly use of bednets through awareness of the disease, but the evaluation below also explains why such ‘awareness’ alone is insufficient.

The USAID Communications and Malaria Initiatives in Tanzania (COMMIT) [23] as a behaviour change communication program was implemented between 2008 and 2012 that incorporated elements of the Theory of Planned Behaviour. The literature states it sought to increase perceptions that bednets are the socially accepted approach for avoiding malaria, foster peoples' confidence in their ability to use bednets every night and improve the attitude that malaria is an unavoidable and constant presence in people's lives. The programme sought to engage communities and individuals with common sets of messages and information through a variety of mutually reinforcing sources, including mass media, rural community outreach, and community-initiated approaches.

The programme’s initial evaluation demonstrated that exposure to the activities improved the self-efficacy necessary to take action to prevent malaria. Nearly 77% of those exposed to the programme put all their children under bednets the previous night, as opposed to 34.6% of those unexposed. Exposure to the campaign significantly increased the perception that nets are effective in stopping malaria and the belief that nets are useful and easy to use.

The subsequent COMMIT Project Performance Evaluation of the impact of the programme commented on the increase in bednet use and suggested that of course awareness of the disease was heightened:

*“(…), communication operates by indirect as well as direct effect. Especially when a behaviour starts becoming the norm, those not directly exposed can still be influenced by what they see and hear around them; direct exposure is not needed in order to be affected by the campaign.”*

*“Common sense, consistent testimony from people on the ground (…) suggest that the millions of print materials, thousands of radio spots and community-level events, (…) have had a significant impact on the malaria behaviours of many Tanzanians.”*

But the evaluation also commented that elimination of malaria demands more than just putting children under a bednet the previous night. It demands a long-term commitment. To eliminate malaria, the task of such elimination must be viewed as a priority, and it is doubtful the Tanzanians would have come to view the task as such merely as a result of this programme. The Executive Summary of the evaluation concluded with the following comment:

*“(…) it is generally accepted that Behaviour Change Communication is an essential, but not sufficient, component of any public health initiative. It is not enough to provide commodities and services: one also has to educate the public about their use and importance and build a continuing demand for them.”*

And the Project Performance Evaluation interestingly concluded with a following recommendation:

*“Awareness levels on key malaria messages are now very high. People understand the dangers of malaria and are familiar with the steps needed to prevent or treat it; however, action needs to be focused on converting that increased awareness into actual behaviour change. A more strategic phase of behaviour change work needs to occur that looks at each desired behaviour, particularly those that have been resistant to change, to better understand the barriers to achieving high levels of compliance with malaria recommendations.”*

Kligler’s education was more than just awareness. His education explained the disease so that the inhabitants understood why the disease would return if certain tasks were not undertaken by them. The inhabitants realised they each had a contribution to make. Further, Kligler would have arranged for each inhabitant to be trained to carry out the maintenance work to ensure it was carried out correctly and thoroughly. Kligler’s education ensured malaria-elimination was a priority.

## Conclusion

It must be remembered that Kligler was an idealistic Jewish Zionist. He had commented that: *“(…) unless something was done to check the ravages of malaria, the reconstruction of Palestine would be a costly if not altogether an impossible effort.”* [24]. The problem of malaria was therefore treated as a priority 100 years ago by the Zionists, otherwise the dream of a Jewish homeland in Palestine would have remained just that, namely just a dream.

Therefore, Kligler brought with him an attitude, namely, that he could not afford to fail in his fight against malaria. He had to make Palestine habitable. The handful of inhabitants in Palestine 100 years ago, Arabs and Jews, all treated this fight against malaria in the same way as a priority thanks to the individual education, and which lent resolve in the fight against the disease.

But what damage did the League of Nations’ 1925 criticism actually do to malaria elimination? Wouldn’t education have received less attention anyway after WW2 with the advent of DDT and the other insecticides? We will never know for sure, because as the WHO historical note comments:

*“The focus of vector control on insecticides meant that environmental management and other alternative methods were underexploited **or even forgotten**."*

Kligler felt confidence in his method and ignored the League of Nation’s criticism, and continued with his method, malaria being eventually declared eliminated in 1967.

It is likely countries may have been discouraged by the criticism from following or even studying Kligler’s approach. Had they done so, these countries would also have become aware of the fight against malaria being conducted as a priority, and which attitude today is generally seen as missing in many malaria-stricken governments around the world. This is a huge missing component.

The world has been denied knowledge of the existence of Kligler and of his work. There were many lessons to be learnt from Kligler’s method and approach. Regretfully, the League of Nations was successful in tossing these lessons into the bin of obscurity.
